# Dual Therapy with Liraglutide and Ghrelin Promotes Brain and Peripheral Energy Metabolism in the R6/2 Mouse Model of Huntington’s Disease

**DOI:** 10.1038/s41598-018-27121-w

**Published:** 2018-06-12

**Authors:** Ana I. Duarte, Marie Sjögren, Maria S. Santos, Catarina R. Oliveira, Paula I. Moreira, Maria Björkqvist

**Affiliations:** 1 CNC - Center for Neuroscience and Cell Biology, University of Coimbra, Coimbra, Portugal; 2 Institute for Interdisciplinary Research (IIIUC), University of Coimbra, Coimbra, Portugal; 3 Brain Disease Biomarker Unit, Department of Experimental Medical Sciences, Wallenberg Neuroscience Center, Lund University, Lund, Sweden; 4 Life Sciences Department, Faculty of Sciences and Technology, University of Coimbra, Coimbra, Portugal; 5 Laboratory of Biochemistry, Faculty of Medicine, University of Coimbra, Coimbra, Portugal; 6 Laboratory of Physiology, Faculty of Medicine, University of Coimbra, Coimbra, Portugal

## Abstract

Neuronal loss alongside altered energy metabolism, are key features of Huntington’s disease (HD) pathology. The orexigenic gut-peptide hormone ghrelin is known to stimulate appetite and affect whole body energy metabolism. Liraglutide is an efficient anti-type 2 diabetes incretin drug, with neuroprotective effects alongside anorectic properties. Combining liraglutide with the orexigenic peptide ghrelin may potentially promote brain/cognitive function in HD. The R6/2 mouse model of HD exhibits progressive central pathology, weight loss, deranged glucose metabolism, skeletal muscle atrophy and altered body composition. In this study, we targeted energy metabolism in R6/2 mice using a co-administration of liraglutide and ghrelin. We investigated their effect on brain cortical hormone-mediated intracellular signalling pathways, metabolic and apoptotic markers, and the impact on motor function in HD. We here demonstrate that liraglutide, alone or together with ghrelin (subcutaneous daily injections of 150 µg/kg (ghrelin) and 0.2 mg/kg (liraglutide), for 2 weeks), normalized glucose homeostatic features in the R6/2 mouse, without substantially affecting body weight or body composition. Liraglutide alone decreased brain cortical active GLP-1 and IGF-1 levels in R6/2 mice, alongside higher ADP levels. Liraglutide plus ghrelin decreased brain insulin, lactate, AMP and cholesterol levels in R6/2 mice. Taken together, our findings encourage further studies targeting energy metabolism in HD.

## Introduction

Huntington’s disease (HD) is an autosomal dominant polyglutamine expansion disease^1^, associated with neuronal loss^2^. In addition, HD is associated with peripheral pathology, including weight loss and muscle atrophy^3–5^. Energy metabolism alterations in HD are likely to contribute to neurodegenerative processes^6^. Mitochondrial dysfunction and widespread changes in energy metabolism have been suggested as underlying key players in HD pathogenesis^7^. Studies in HD models indicate that mutant huntingtin (HTT) disrupts mitochondrial bioenergetics and adenosine triphosphate (ATP) generation^8,9^. A dysfunctional central energy metabolism is thought to promote neurodegenerative processes in HD^10^. This is in line with growing evidence demonstrating that many neurodegenerative disorders are metabolic diseases, mediated by impairments in brain energy metabolism, including insulin responsiveness and glucose utilization (reviewed in^11,12^).

Therefore, targeting mechanisms underlying metabolic alterations could be beneficial, not only for metabolic features, but also for neuronal function.

Peripheral and central energy metabolism are affected by gut peptide hormones, such as glucagon-like peptide-1 (GLP-1) and ghrelin (reviewed in^13^). GLP-1 is a key determinant of blood glucose homeostasis, due to its ability to slow down gastric emptying and enhance pancreatic insulin secretion^14^. Ghrelin exerts metabolic effects throughout the body and increases body weight by enhancing appetite^15^.

GLP-1 mimetics (such as exendin-4 and liraglutide) have been shown to exert neuroprotective effects, being beneficial in Parkinson’s disease (PD)^16^, Huntington’s disease (HD)^17^ and Alzheimer’s disease (AD)^18^ mouse models, and in clinical studies involving PD and AD patients^19^. Similarly, ghrelin and its analogues have been shown to have neuroprotective effects in PD and AD mouse models^20,21^.

Since HD is associated with weight loss and GLP-1 mimetics have anorectic properties, it is challenging to use GLP-1 mimetics in HD. We therefore hypothesize that peripheral injection of liraglutide, together with the orexigenic peptide hormone ghrelin, would maintain body weight and protect against brain/cognitive dysfunction in HD.

In this study, we investigated metabolic alterations in the R6/2 mouse model of HD. We used a pharmacological approach to determine the effects of subcutaneous (s.c.) co-injection of liraglutide and ghrelin on brain cortical hormone-mediated intracellular signalling pathways, metabolic and apoptotic markers, and the impact on motor-cognitive function in the R6/2 mouse model of HD.

## Results

### Liraglutide alone and in combination with ghrelin normalize peripheral glucose homeostasis in R6/2 mice

Ghrelin has been shown to increase body weight^15^, while liraglutide has been shown to exert the opposite effect^22^. The R6/2 mouse model displays a progressive weight loss. We therefore monitored body weight twice weekly, starting at 9 weeks of age. Subcutaneous administration of vehicle (NaCl), liraglutide alone or in combination with ghrelin was conducted for 2 weeks, starting at 10 weeks of age. As detailed in Material and Methods, at the end of treatment 12-weeks old mice were fasted for ~6 h (starting late in the evening) before euthanasia, and blood and brain cortices collected for remaining measurements. Similar to previous studies, weight loss was present in 12-week old R6/2 mice compared to wildtype (WT) littermates (Supplementary Fig. 1a). Except for the 1.4-fold lower fat mass composition induced by liraglutide *per se*, the 2-week treatment strategy chosen did not affect R6/2 mouse body weight or body composition at 12 weeks of age (Supplementary Fig. 1).

Food consumption was assessed daily during 14 days, from 11 weeks of age in an additional treatment group. Food consumption was not different comparing R6/2 treated with liraglutide in combination with ghrelin with R6/2 mice treated with only liraglutide. Food consumption differed compared to WT mice treated with vehicle the first 2 days of the 2-week treatment period and on day 12 and 14 (see Supplementary Fig. 2 for body weight and food consumption).

Both liraglutide and ghrelin have been shown to affect glucose homeostasis^13^. We therefore evaluated the effect of liraglutide alone and in combination with ghrelin on glucose homeostasis parameters in 12-week old R6/2 mice and WT littermates (Table 1). In this study, R6/2 mice exhibited increased serum glucose levels, in accordance with previous findings^23^. Interestingly, both liraglutide alone and together with ghrelin normalized serum glucose levels in 12-week old R6/2 mice (by 1.7- and 1.5-fold, with *P* = 0.0019 and *P* = 0.0136, respectively). HOMA-IR and HOMA-β indexes (homeostasis models to assess insulin resistance and β-cell function, respectively)^24,25^ were also calculated (Table 1). HOMA-β was significantly decreased in 12-week old R6/2 mice compared to WT littermates, which was rescued after treatment with liraglutide alone and in combination with ghrelin (by a 2.7- and 3.5-fold increase, with a *P* = 0.0452 and *P* = 0.004, respectively) (Table 1). HOMA-IR was significantly increased in 12-week old R6/2 mice compared to WT littermates, which was normalized after treatment with liraglutide alone and in combination with ghrelin (by a 1.7- and 1.4-fold increase, respectively, with a *P* = 0.0074 and *P* = 0.0298) (Table 1).

**Table 1:** Effect of liraglutide plus ghrelin on blood biochemical features in 12-week old R6/2 mice.

	WT mice	R6/2 mice		
	+NaCl	+NaCl	+Liraglutide	+Liraglutide +Ghrelin
**Serum glucose levels** (mM, n = 10)	9.3 ± 0.47	15.11 ± 1.27**	8.72 ± 0.89^££^	9.89 ± 1.6^£^
**Serum insulin levels** (mg/L, n = 8–10)	0.079 ± 0.0003	9.3 ± 0.47	15.11 ± 1.27**	0.081 ± 0.0011
**HOMA-IR** (n = 10)	0.81 ± 0.04	1.33 ± 0.11**	0.80 ± 0.095^££^	0.99 ± 0.15^£^
**HOMA-β** (n = 9)	660.8 ± 36.8	339.8 ± 31.1*	927.1 ± 180.6^£^	1202 ± 357.6^££^

Data are mean ± SEM of the indicated number of mice/group. Statistical significance: *P < 0.05, **P < 0.01 *vs*. saline-treated WT mice; and ^£^*P* < 0.05, ^££^*P* < 0.01 *vs*. saline-treated R6/2 mice, by one-way ANOVA, with Tukey or Sidak post-hoc tests for multiple comparisons (for a Gaussian distribution), or with the Kruskal-Wallis test, with Dunn post-test (non-Gaussian distribution).

### Liraglutide in combination with ghrelin moderately affects R6/2 mouse brain cortical intracellular signalling

**Figure 1:**
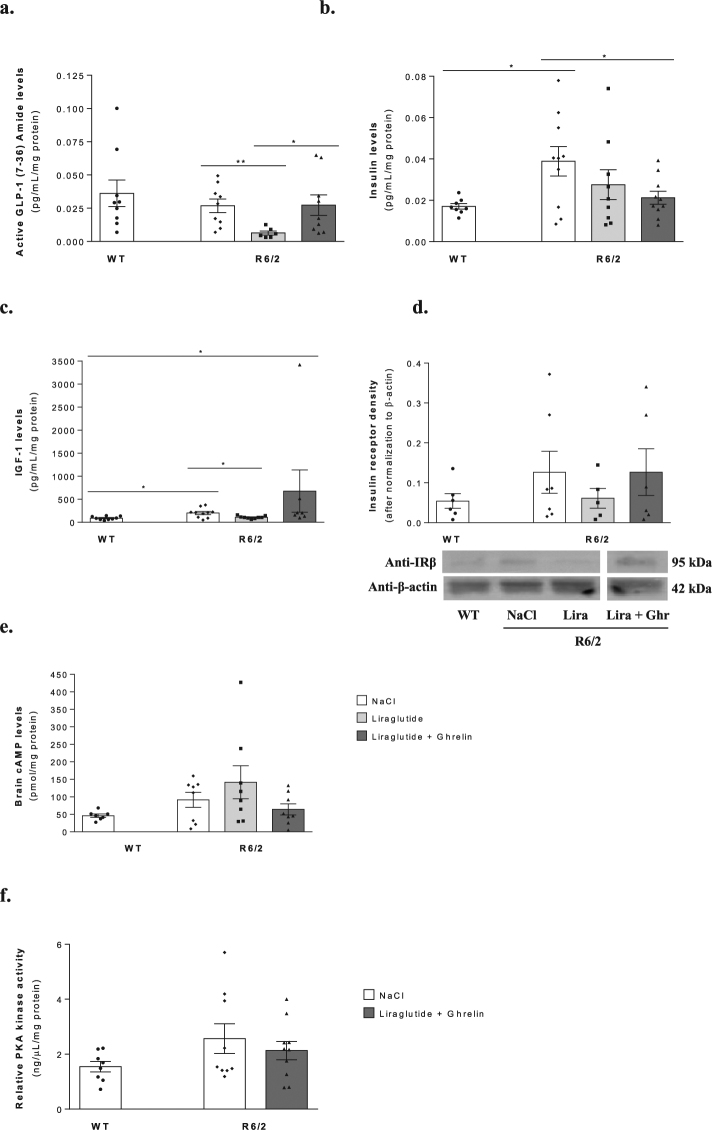
Effect of liraglutide plus ghrelin administration on R6/2 mouse brain cortical GLP-1, insulin and IGF-1 levels and pivotal downstream signalling pathways. Brain cortical levels of GLP-1 (**a**), insulin (**b**) and IGF-1 (**c**), IRβ density (**d**), cAMP levels (**e**) and PKA activity (**f**). The increased cortical insulin levels found in R6/2 mice compared to WT littermates were reduced after liraglutide and ghrelin treatment (**b**), while liraglutide alone normalized the IGF-1 levels (**c**). Data are mean ± SEM of the indicated number of mice/group. Statistical significance: *P < 0.05, by one-way ANOVA, with Tukey or Sidak post-hoc tests for multiple comparisons (for a Gaussian distribution) (**b**), or with the Kruskal-Wallis test, with Dunn post-test (non-Gaussian distribution) (**c**). [Figure a: n = 9 WT, n = 9 R6/2, n = 6 R6/2 + Lira, n = 9 R6/2 + Lira + Ghr. Figure b: n = 8 WT, n = 10 R6/2, n = 9 R6/2 + Lira, n = 10 R6/2 + Lira + Ghr. Figure c: n = 9 WT, n = 10 R6/2, n = 9 R6/2 + Lira, n = 7 R6/2 + Lira + Ghr. Figure d: n = 6 WT, n = 7 R6/2, n = 5 R6/2 + Lira, n = 6 R6/2 + Lira + Ghr. Figure e: n = 7 WT, n = 8 R6/2, n = 8 R6/2 + Lira, n = 8 R6/2 + Lira + Ghr. Figure f: n = 8 WT, n = 9 R6/2, n = 10 R6/2 + Lira + Ghr].

To assess the possible effects of liraglutide and ghrelin on brain cortical signalling, we assessed cortical levels of GLP-1, insulin, IGF-1 and insulin receptor density. Despite no significant changes in active GLP-1 levels, 2.3- and 2.2-fold higher insulin (*P* = 0.045) and IGF-1 levels (*P* = 0.0045) were found in R6/2 mouse cortex compared to WT littermates (Fig. 1a–c). Despite no significant differences between these cohorts in insulin receptor density, cAMP levels, relative PKA kinase activity or p-ERK1,2 (Fig. 1d–f; Supplementary Fig. 3), we cannot rule out a stimulation of insulin and/or GLP-1 receptors.

Liraglutide administration *per se* decreased R6/2 mouse brain cortical active GLP-1 (4.2-fold, *P* = 0.0094) and IGF-1 levels (1.9-fold, *P* = 0.0045) compared to vehicle treated R6/2 mice (Fig. 1a,c).

Co-administration of liraglutide and ghrelin appeared to exert distinct effects, since the even lower brain insulin levels in R6/2 mice (by 1.8-fold, *P* = 0.045) compared to vehicle treated mice (Fig. 1b) were not followed by significant changes in the remaining hormones levels (Fig. 1a,c).

### Liraglutide in combination with ghrelin moderately affects glycolysis and energy metabolism in R6/2 mouse brain cortex

Brain energy metabolism has been shown to be altered in R6/2 mice^26^. We therefore evaluated the possible effects of liraglutide and ghrelin on tricarboxylic acid cycle- and energy metabolism-related parameters in cortex from R6/2 mice and WT mice.

**Figure 2:**
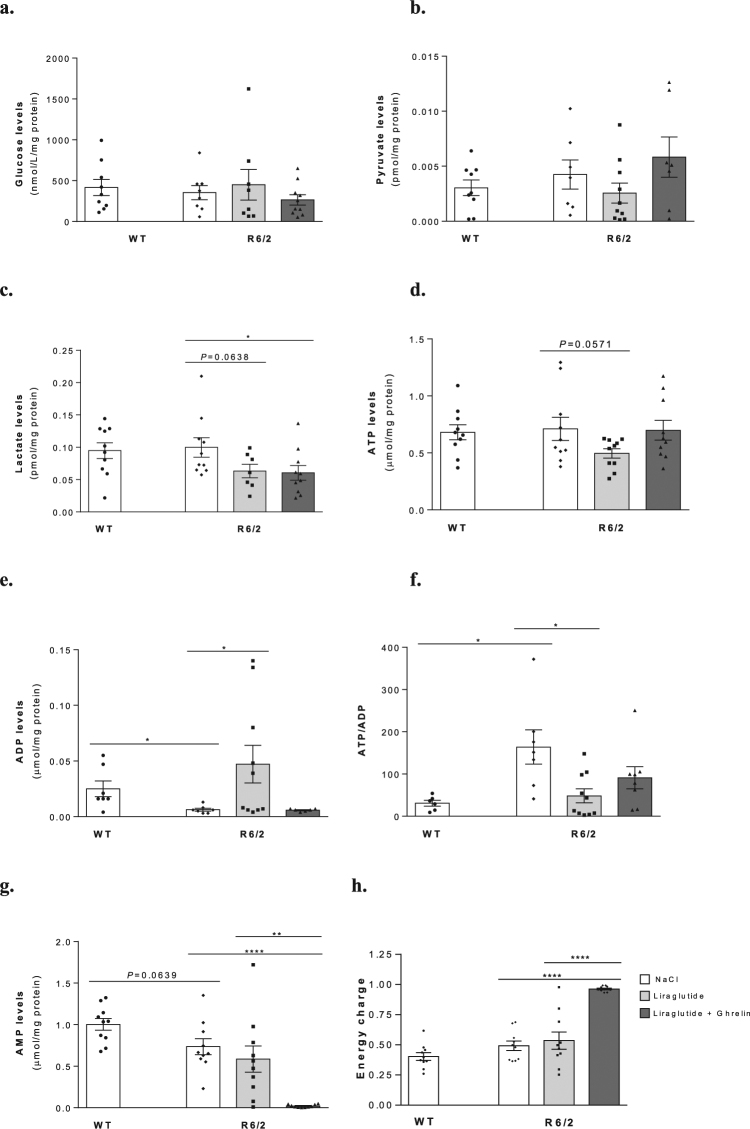
Effect of liraglutide plus ghrelin administration on R6/2 mouse brain cortical levels of glucose, markers for glycolysis, anaerobic metabolism and energy formation. Brain cortical levels of glucose (**a**), pyruvate (**b**), lactate (**c**), ATP (**d**), ADP (**e**), ATP/ADP (**f**), AMP (**g**) and energy charge (**h**). ADP level was significantly decreased (**e**), while the ATP/ADP ratio (**f**) was significantly increased in the cortex of 12 weeks old vehicle treated R6/2 mice compared to WT littermates. Liraglutide treatment alone normalized cortical ADP levels and ATP/ADP ratio, while liraglutide together with ghrelin lowered AMP (**g**) and lactate levels (**c**) and increased the energy charge (**h**) in R6/2 mice compared to WT littermates. Data are mean ± SEM of the indicated number of mice/group. Statistical significance: *P < 0.05, **P < 0.01, ****P < 0.0001, by one-way ANOVA, with Tukey, Sidak or unprotected Fisher’s LSD post-hoc tests for multiple comparisons (for a Gaussian distribution) (**a**–**d**,**g**,**h**), or with the Kruskal-Wallis test, with Dunn post-test (non-Gaussian distribution) (e., f). [Figure a: n = 9 WT, n = 8 R6/2, n = 8 R6/2 + Lira, n = 10 R6/2 + Lira + Ghr. Figure b: n = 9 WT, n = 7 R6/2, n = 10 R6/2 + Lira, n = 7 R6/2 + Lira + Ghr. Figure c: n = 10 WT, n = 10 R6/2, n = 7 R6/2 + Lira, n = 10 R6/2 + Lira + Ghr. Figure d: n = 10 WT, n = 10 R6/2, n = 10 R6/2 + Lira, n = 10 R6/2 + Lira + Ghr. Figure e: n = 7 WT, n = 7 R6/2, n = 10 R6/2 + Lira, n = 6 R6/2 + Lira + Ghr. Figure f: n = 6 WT, n = 7 R6/2, n = 10 R6/2 + Lira, n = 8 R6/2 + Lira + Ghr. Figure g: n = 10 WT, n = 10 R6/2, n = 10 R6/2 + Lira, n = 10 R6/2 + Lira + Ghr. Figure h: n = 10 WT, n = 10 R6/2, n = 10 R6/2 + Lira, n = 10 R6/2 + Lira + Ghr].

In this study, R6/2 mice exhibited a 4-fold decrease in ADP levels (*P* = 0.0234) along with a 5.3-fold increase in ATP/ADP ratio (*P* = 0.0316), which were both normalized by 2 weeks of liraglutide administration (*P* = 0.0221 and *P* = 0.026, respectively) (Fig. 2d,f). Despite no significant changes in brain cortical glucose, pyruvate, lactate, ATP or AMP levels in vehicle treated R6/2 mice compared to WT littermates (Fig. 2a–d, g,h), co-administration of liraglutide and ghrelin induced a 1.6-fold and a 35-fold decrease in both lactate (*P* = 0.03) and AMP (*P* < 0.0001) levels (Fig. 2c,g), and improved the brain energy status by 2-fold (P < 0.0001) (Fig. 2h), suggesting a decrease in cortical catabolic pathways.

### Liraglutide in combination with ghrelin normalize brain triglyceride, and cholesterol levels in R6/2 mice

**Figure 3:**
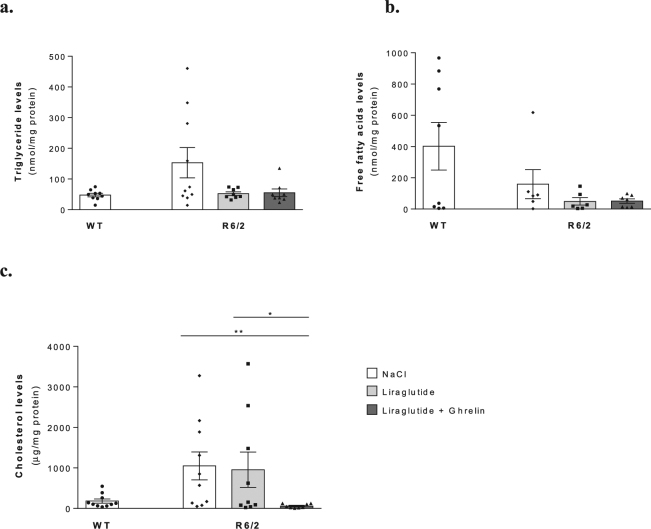
Liraglutide plus ghrelin decreases brain triglyceride, FFA and cholesterol levels in R6/2 mice. Effect of co-administration of liraglutide together with ghrelin on R6/2 mouse brain cortical triglycerides, free fatty acids and cholesterol levels. Brain cortical levels of triglycerides (**a**), free fatty acids (**b**) and cholesterol (**c**). Cholesterol levels were lowered by 2 weeks of liraglutide and ghrelin treatment in cortex of 12 weeks old R6/2 mice compared to WT littermates. Data are mean ± SEM of the indicated number of mice/group. Statistical significance: *P < 0.05, **P < 0.01, by the Kruskal-Wallis test, with Dunn post-test (non-Gaussian distribution). [Figure a: n = 9 WT, n = 10 R6/2, n = 8 R6/2 + Lira, n = 8 R6/2 + Lira + Ghr. Figure b: n = 8 WT, n = 6 R6/2, n = 6 R6/2 + Lira, n = 7 R6/2 + Lira + Ghr. Figure c: n = 10 WT, n = 10 R6/2, n = 9 R6/2 + Lira, n = 8 R6/2 + Lira + Ghr].

The cholesterol biosynthetic pathway has been shown to be impaired in HD, with alterations in cholesterol and triglycerides levels^27^. Regarding the possible use of triglycerides, free fatty acids (FFA) and cholesterol as alternative brain metabolic substrates, we observed that, although not significant, there was a trend towards a change in brain cortical triglyceride, cholesterol and FFA levels. Cortical triglyceride and cholesterol levels were ~3- and ~5-fold higher (*P* = 0.1555 and *P* = 0.0985) in saline-treated R6/2 mice, whereas their brain FFA were ~3 times lower (*P* = 0.8611) than in WT littermates (Fig. 3a–c). Liraglutide *per se* or upon co-administration with ghrelin normalized brain triglyceride and cholesterol levels to nearly those of WT mice (Fig. 3a,c). These results suggest that liraglutide, alone or co-administrated with ghrelin, may attenuate the use of triglycerides and cholesterol as brain alternative metabolites.

### Effect of liraglutide in combination with ghrelin on R6/2 mouse brain cortical apoptotic pathways

Low levels of brain caspase activity have been shown to affect axonal function^28^, and cognitive deficits^29^, whereas increased caspase activity may lead to inhibition of autophagy^30,31^. We therefore investigated the effects of liraglutide and ghrelin on caspase activities in R6/2 and WT mice brain cortex.

**Figure 4:**
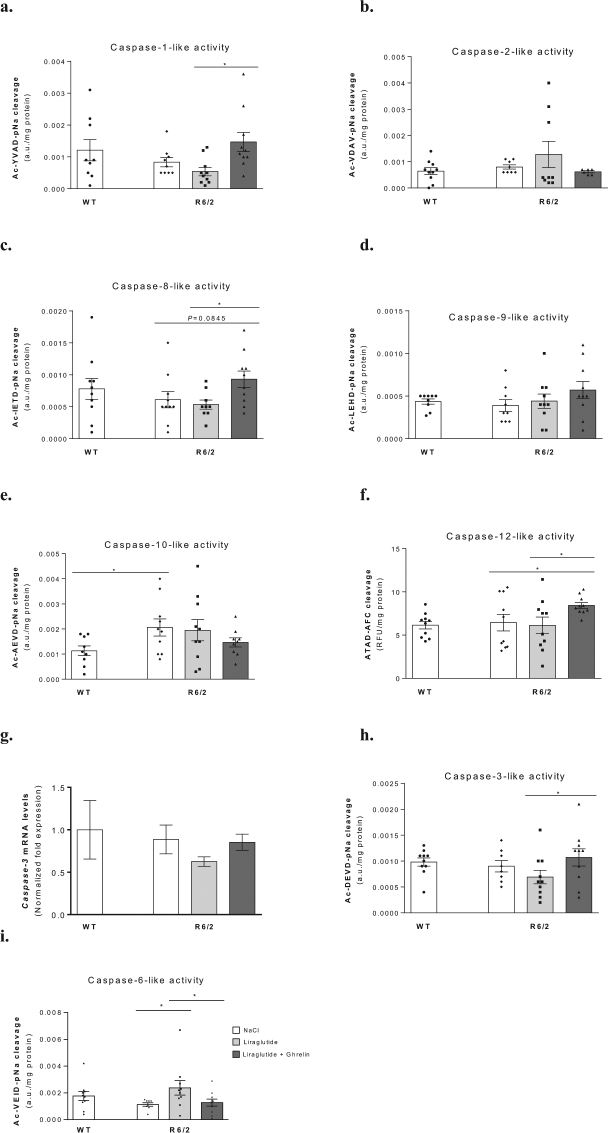
Effect of liraglutide plus ghrelin administration on R6/2 mouse brain cortical initiator and effector caspases activities. Brain cortical activities of caspases-like-1 (**a**), -2 (**b**), -8 (**c**), -9 (**d**), -10 (**e**), -12 (**f**), mRNA levels of caspase-3 (**g**), and caspase-3-like (**h**) and −6 activities (**i**). A significant increase was found in Caspase-10-like activity (**e**) in cortex of 12 weeks old vehicle treated R6/2 mice compared to WT littermates; however, no improvement was found with either liraglutide alone or together with ghrelin. Despite no significant alterations in R6/2 mice, Caspase-6-like activity (**i**) was stimulated by liraglutide alone, while together with ghrelin there was a stimulation of Caspase-12-like activity (**f**) in cortex of R6/2 mice. Data are mean ± SEM of the indicated number of mice/group. Statistical significance: *P < 0.05, by the Kruskal-Wallis test, with Dunn post-test (non-Gaussian distribution) (**a**,**b**,**d**,**f**,**i**), or by the one-way ANOVA, with Tukey, Sidak, Bonferroni or unprotected Fisher’s LSD post-hoc tests for multiple comparisons (for a Gaussian distribution) (**c**,**e**,**h**). [Figure a: n = 9 WT, n = 9 R6/2, n = 10 R6/2 + Lira, n = 10 R6/2 + Lira + Ghr; Figure b: n = 10 WT, n = 8 R6/2, n = 9 R6/2 + Lira, n = 6 R6/2 + Lira + Ghr. Figure c: n = 10 WT, n = 10 R6/2, n = 9 R6/2 + Lira, n = 10 R6/2 + Lira + Ghr, Figure d: n = 9 WT, n = 9 R6/2, n = 10 R6/2 + Lira, n = 10 R6/2 + Lira + Ghr. Figure e: n = 9 WT, n = 10 R6/2, n = 10 R6/2 + Lira, n = 9 R6/2 + Lira + Ghr. Figure f: n = 10 WT, n = 10 R6/2, n = 10 R6/2 + Lira, n = 10 R6/2 + Lira + Ghr. Figure g: n = 6 WT, n = 6 R6/2, n = 6 R6/2 + Lira, n = 6 R6/2 + Lira + Ghr. Figure h: n = 10 WT, n = 8 R6/2, n = 10 R6/2 + Lira, n = 10 R6/2 + Lira + Ghr. Figure i: n = 10 WT, n = 8 R6/2, n = 10 R6/2 + Lira, n = 10 R6/2 + Lira + Ghr].

A 2-fold increase in brain cortical Caspase-10-like activity was found in vehicle treated R6/2 compared to WT mice, while no significant changes were observed for the other initiator Caspases-1-, -2-, -8-, -9-like activities (Fig. 4a–f). These may account for unchanged Caspase-3 mRNA and Caspase-3 enzyme activity (Fig. 4g,h), and Caspase-6-like activity (Fig. 4i) under these conditions. The 2-fold stimulation of Caspase-6-like activity induced by liraglutide per se in R6/2 mouse brain (Fig. 4i) was not accompanied by significant changes in the remaining Caspase-like activities or mRNA under these conditions.

Co-administration of liraglutide plus ghrelin resulted in a 1.3-fold stimulation of brain cortical Caspase-12-like activity in R6/2 mice (Fig. 4c,f) that, nonetheless, had no significant impact on the activities of the effector Caspases-3 and -6-like (Fig. 4h,i).

### Liraglutide in combination with ghrelin does not affect motor and exploratory activities in R6/2 mouse

**Figure 5:**
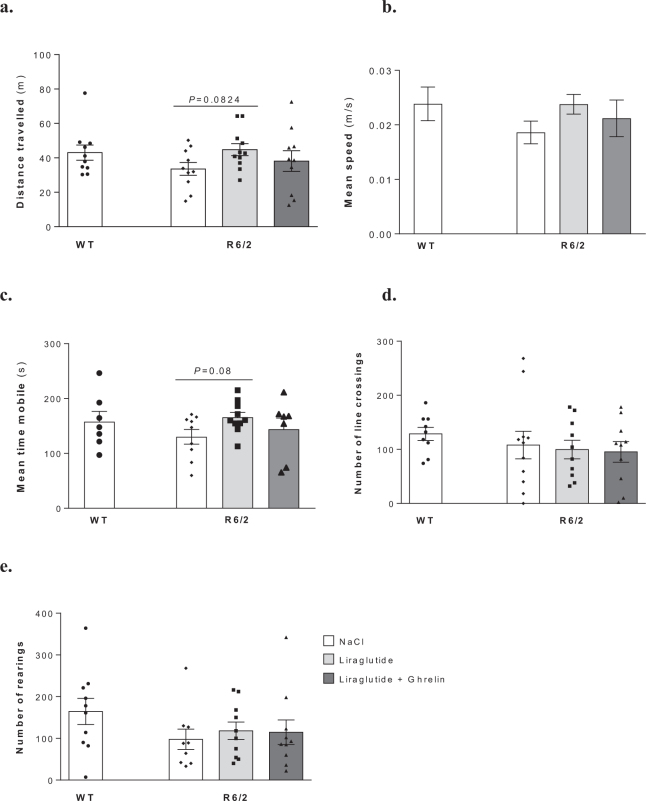
Effect of liraglutide plus ghrelin administration on R6/2 mouse exploratory and locomotor behavior. Distance travelled (**a**), mean speed (**b**), mean time mobile (**c**), number of line crossings (**d**) and rearings (**e**). No significant exploratory and locomotor behavior was seen in 12 weeks old R6/2 mice compared to WT littermates, and no significant improvement was seen after 2 weeks administration of either liraglutide alone or together with ghrelin. Data are mean ± SEM of the indicated number of mice/group. Statistical significance was given by the one-way ANOVA, with unprotected Fisher’s LSD post-hoc tests for multiple comparisons (for a Gaussian distribution) (**a**). [Figure a: n = 10 WT, n = 10 R6/2, n = 11 R6/2 + Lira, n = 10 R6/2 + Lira + Ghr. Figure b: n = 7 WT, n = 9 R6/2, n = 10 R6/2 + Lira, n = 7 R6/2 + Lira + Ghr. Figure c: n = 7 WT, n = 8 R6/2, n = 10 R6/2 + Lira, n = 7 R6/2 + Lira + Ghr. Figure d: n = 9 WT, n = 11 R6/2, n = 10 R6/2 + Lira, n = 10 R6/2 + Lira + Ghr. Figure e: n = 10 WT, n = 9 R6/2, n = 10 R6/2 + Lira, n = 10 R6/2 + Lira + Ghr].

Motor deficits along with a clasping behaviour phenotype are main features of HD mouse models, which progress with the disease^32,33^. However, in this study we could not find any significant differences between experimental groups in terms of the locomotor and exploratory activity parameters evaluated during the open-field test (Fig. 5a–e), nor in R6/2 mice after liraglutide and ghrelin co-administration (Fig. 5a,c). However, an increase in paw clasping to a score of 2 was observed in vehicle-treated R6/2 mice compared with WT mice (Table 2) that, nonetheless, was not significantly changed by liraglutide alone or in combination with ghrelin (Table 2).

**Table 2:** Effect of peripheral liraglutide plus ghrelin co-injection on paw clasping scores in R6/2 mice.

	WT +NaCl	R6/2 +NaCl	R6/2 +Liraglutide	R6/2 +Liraglutide +Ghrelin
Paw clasping score	0.5	2**	2	1

Paw clasping phenotype was scored upon mouse suspension by the tail for 180 s. A score of 0 represents no clasping behaviour, 1 occurs when the hind paws touch each other for at least 1 s, and 2 mean that hind paws clasp for 5 s or more. R6/2 mice showed a paw clasping phenotype at 12 weeks of age compared to WT littermates. However, no significant improvement was seen with 2 weeks administration of either liraglutide alone or together with ghrelin. Data are medians of 10 animals/group, as described in Materials and Methods. **P < 0.01 *vs*. saline-treated WT mice, by the Kruskal-Wallis test, with Dunn post-test (non-Gaussian distribution).

## Discussion

Accumulating evidence over the last decade supports the concept of HD being a metabolic disorder. Animal studies have demonstrated that targeting peripheral energy metabolism might have beneficial effects on both central and peripheral pathology in HD^3,17^. HD is associated with progressive weight loss and a lower body mass index (BMI), and a higher BMI has been shown to correlate with slower disease progression^34^. As a catabolic state is present in human HD^35,36^ and HD mouse models^32^, this suggests that normalization of energy metabolism might be beneficial.

The R6/2 mouse model is the most widely used model of HD^32^, replicating many features seen in human patients, including weight loss due to increased energy metabolism^37^. We recently showed that chronic ghrelin administration for 4 weeks postpones R6/2 mouse weight loss by one week^38^. Administration of the GLP-1 analogue, liraglutide, has been shown to be associated with weight loss^22,39^, which could be problematic if used in a mouse model displaying weight loss, such as the R6/2 mouse. However, we here demonstrate that daily injections of either liraglutide alone or together with ghrelin for 2 weeks (from 10 to 12 weeks of age) did not further decrease body weight in R6/2 mice. Body composition alterations seen in R6/2 mice^40^, such as lower fat mass, was further decreased with liraglutide administration alone, while no changes were found in liraglutide and ghrelin treated R6/2 mice.

The choice of a 2-week treatment with ghrelin and liraglutide was based on our previous study on the effects of a 2- and a 4-week treatment with ghrelin in R6/2 mice skeletal muscle^38^. Although a 2-week administration of ghrelin did not change R6/2 mice body weight or their peripheral type 2 diabetes features^38^, this was sufficient to reverse the expression of catabolic genes (e.g. *Caspase 8*, *Creb1* and *Traf-5)* and muscle morphology, as well as to rescue their nest building defects^38^.

Metabolic effect of drug treatment can of course be related to possible alterations in food intake. It has previously been shown that R6/2 mice display increased food intake in comparison to WT littermates^37^. In our 2-week study there was no change in food intake comparing treated groups, however a decreased food intake in comparison to WT littermates could be noted during a few days of treatment period. Since food consumption was similar the majority of days, food consumption-induced differences should be marginal. However, of course this should be considered when interpreting results.

Although hyperglycaemia along with reduced insulin levels has been shown previously in R6/2 mice^23^, in the present study we found that elevated levels of serum glucose were accompanied by normal insulin levels, increased HOMA-IR and decreased HOMA-β in R6/2 mice compared to WT littermates. This may be explained by the fact that our R6/2 mice presented an early to middle stage disease phenotype and, thus, insulin production was not dramatically affected yet. This was in line with previous studies demonstrating a variation in disease progression depending on the CAG repeat size^41,42^.

Activation of the GLP-1 receptor by liraglutide and other analogues, such as exendin-4, has been shown to exert anti-diabetic effects, by improving pancreatic β-cell function and glucose regulation in HD mouse models^17,43^. Conversely, studies involving the effect of ghrelin administration on insulin secretion β-cell in other rodent and *in vitro* models have given contradictory results, with both inhibitory^44^ and beneficial effects^45^. In the present study, liraglutide injected alone or together with ghrelin normalized peripheral basal glucose levels and both HOMA-β and HOMA-IR (two mathematical models widely used to evaluate β-cell function and insulin resistance, respectively, from fasting glycemia and insulin or C-peptide levels^24,25^) in R6/2 mice, suggesting a beneficial effect on glucose homeostasis, insulin resistance and pancreatic β-cell function.

Mitochondrial dysfunction and oxidative stress have been shown to contribute to R6/2 mouse central pathology, probably resulting from elevated insulin/IGF-1 signalling pathways and subsequent progression of neurodegeneration^46^. Conversely, others have suggested that a compensatory increase in brain insulin levels may overcome systemic insulin resistance in R6/2 mice^47^. In line with the observed peripheral insulin resistance, elevated brain insulin levels in our R6/2 mice were normalized after 2 weeks of co-administration of liraglutide and ghrelin. Decreased brain cortical active GLP-1 and IGF-1 levels after liraglutide administration in R6/2 mice suggested that an activation of compensatory mechanisms could occur to overcome their lower brain hormone levels and maintain downstream cAMP- and/or ERK1,2-mediated signalling pathways.

The unchanged brain cortical ATP levels in vehicle treated R6/2 mice was in line with previous studies in HD mice^48–50^ and early stage HD patients^51^. This reinforced the idea that, though mutant HTT may hamper brain glycolysis, it may not directly affect mitochondrial oxidative phosphorylation, thus preserving ATP synthesis^48,49^. Since the later depends mostly on ADP levels, the maintenance of ATP levels in vehicle treated R6/2 mouse may occur at the expense of brain ADP and AMP levels^52^. This may be accompanied by an increment in total creatine levels (creatine plus phosphocreatine, an alternative source of ATP)^52^. Of note, Mochel *et al*.^24^ found that striatal ATP and phosphocreatine levels were inversely correlated with the number of CAG repeats in HD mice. Alternatively, we cannot exclude that the decreased brain ADP and AMP in vehicle treated R6/2 mice may arise from the maintenance of their cAMP and/or of adenosine levels, a critical molecule in neurotransmission and energy metabolism, whose increased extracellular content was beneficial in R6/2 mouse brains^53^. More recently, Kao *et al*. (2016)^54^ suggested that a compensatory biphasic regulation of striatal adenosine tone in HD rodents may arise with disease progression. This may be accompanied by an increased expression and activity of striatal adenosine kinase in late-stage R6/2 mice, ultimately converting adenosine into AMP or inosine and thereby decreasing its levels^54^. Additionally, the tendentiously lower brain cortical lactate and ATP levels, and higher ADP levels associated with subcutaneous liraglutide treatment in R6/2 mice suggested that it may not only partially inhibit their brain glycolysis and energy metabolism, but the lower amount of ATP formed may be also used to further yield ADP and cAMP, thus maintaining their intracellular pools.

Caspases are pivotal players in both apoptosis and autophagy, controlling the turnover of protein aggregates and elimination of damaged cells^55^. However, Caspases-3 and -6 were also found to play other non-apoptotic roles in the CNS^56^. The significant increase in brain Caspase-10-like activity of vehicle treated R6/2 mice compared to WT littermates was not significantly affected by either liraglutide alone or in combination with ghrelin. Despite no significant changes in Caspase-6-like or Caspase-12-like activities in vehicle treated R6/2 mice, liraglutide alone significantly increased Caspase-6-like activity, and in combination with ghrelin stimulated Caspase-12-like activity in R6/2 mice. This could indicate that both liraglutide alone or co-administered with ghrelin may exert some neuroprotection in HD. Unchanged Caspase-3 mRNA expression, and Caspase-3 and -6-like activity suggests that caspase-mediated apoptosis may not be involved in R6/2 mouse neuronal death and locomotor deficits.

Although R6/2 mice display progressive motor dysfunction^30,31^, there were no effects on their motor dysfunction in the present study, evaluated by open field and paw clasping behaviour testing. This could have several reasons but, most likely, the short treatment time was not long enough to translate into functional motor-cognitive recovery. Hence, further studies using longer treatment strategies, possibly starting at an earlier age, are encouraged.

In conclusion, our data demonstrate beneficial effects of liraglutide alone and in combination with ghrelin on peripheral glucose homeostasis in R6/2 mice. Co-administration of liraglutide and ghrelin decreased brain cortical insulin, lactate, AMP and cholesterol levels in R6/2 mice, while liraglutide alone decreased brain cortical active GLP-1 and IGF-1 levels in R6/2 mice, alongside higher ADP levels.

Our results support further studies targeting (brain) energy metabolism, which might exert beneficial effects on HD progression. Evaluating underlying effects of liraglutide and ghrelin administration in HD are warranted.

## Material and Methods

### Animals

All experimental procedures performed on mice were carried out in accordance with the approved guidelines in the ethical permit approved by The Malmö/Lund Animal Welfare and Ethics Committee (ethical permit number: M5-15).

Male transgenic R6/2 HD mice (expressing exon 1 of the HD gene)^33^ and their wild-type (WT) littermates were used. Mice were obtained through crossing heterozygous R6/2 males with WT Females (F1 of CBAxC57BL/6 J). Tail tips were sent on dry ice to Laragen (Laragen Inc., CA, USA) for CAG repeat length determination, by polymerase chain reaction assay. The R6/2 mice used in this study had a CAG repeat size ranging from 275–312. The CAG repeat size of our R6/2 colony, results in a disease progression slower than that of the R6/2 mouse with 150 CAG repeats, as described by Morton and co-workers in 2009^57^. In our colony, 18-week old R6/2 mice correspond to overt disease (with robust changes in CSF Neurofilament light chain levels and striatal volume and body weight changes^58^), and 12-week old R6/2 mice correspond to late premanifest disease (similar to what is described by Morton *et al*.^57^.

Mice were housed in groups with *ad libitum* access to chow food and water under standard conditions (12 h light/dark cycle, 22 °C).

Ghrelin (Rat, mouse; 100 µL, 150 μg/kg; Phoenix Pharmaceuticals, Belmont) and/or liraglutide [(Lys(γ-Glu-palmitoyl)26,Arg34)-GLP-1 (7-37)]; 100 µL; 0.2 mg/kg; Bachem, Bubendorf) or sterile NaCl (vehicle; 100 µL) was injected subcutaneously (s.c.) once daily, for 2 weeks, from the age of 10 weeks onwards (prior to R6/2 weight loss). 150 µg/kg ghrelin has previously been shown to exert beneficial effects on body weight^59^. Body weight was monitored twice/week, commencing one week prior to ghrelin and liraglutide administration.

Food consumption was monitored daily from 11 weeks of age (mice grouped per genotype and 1–3 animals per cage) during 2 weeks of ghrelin and liraglutide administration, by measuring the difference between the pre-weighed chow food and the weight of chow after 24 h. Mice had *ad libitum* access to water and food.

At the end of the 2-week treatment, mice were fasted for ~6 h (starting late in the evening) and afterwards body composition was measured using the Lunar Prodigy dual energy x-ray absorptiometry (DEXA; GE Lunar Corp., Madison, WI) and thereafter mice were euthanized, blood was collected and brain was dissected. Tissue samples were snap-frozen in liquid nitrogen and stored at −80 °C until further use. Serum obtained from blood samples was collected by centrifugation at 2000 × *g*, for 10 min, at 4 °C, and immediately frozen to −80 °C.

### Serum Analyses

Serum glucose levels were measured using the glucose oxidase method (Infinity Glucose Hexokinase Kit, Thermo Scientific, Middletown, VA, USA), and levels of insulin were determined by the Mercodia Mouse Insulin ELISA Kit (Uppsala, Sweden). The homeostatic model assessment (HOMA) was calculated to measure insulin resistance (IR) and pancreatic beta-cell function (β). HOMA-IR was calculated as follows: (Insulin X Glucose)/22.5, and HOMA-β as follows: (20 × Insulin)/(Glucose − 3.5)^24^.

### Isolation and Preparation of Brain Cortical Homogenates

Mice were weighed and euthanized by decapitation, and brains were immediately removed. Brain cortices were immediately dissected and snap-frozen for further studies. Immediately before the experiments, brain cortices were homogenized at 0–4 °C in lysis buffer, containing (in mM) the following: 25 HEPES, 2 MgCl_2_, 1 EDTA, 1 EGTA, (pH 7.4), supplemented with 2 mM DTT, 100 μM PMSF, and commercial protease and phosphatase inhibitors cocktails. The homogenate was centrifuged at 17,968 × *g* for 10 min, at 4 °C, in a Sigma 2–16 K centrifuge to remove the nuclei, and the resulting supernatant was collected. The pellet was further resuspended in supplemented buffered solution and centrifuged again at 17,968 × *g* for 10 min, at 4 °C. The supernatant was added to the previously obtained one and protein content was measured using Pierce BCA Assay Kit (Thermo Scientific, Rockford, IL, USA) according to manufacturer’s protocol.

### Quantification of Pivotal GLP-1/Insulin/IGF-1 Signalling Markers’ Levels

Brain cortical GLP-1/Insulin/IGF-1-mediated signalling pathways were analysed by the determination of the pivotal molecules: active GLP-1, insulin, IGF-1 and cAMP using commercially available ELISA kits, according to manufacturers’ instructions with slight modifications.

Active GLP-1 levels were measured in 5 μL of each brain cortical homogenate (working dilution of 1:5) by the Fluorescent ImmunoAssay kit for Active GLP-1 (7-36) Amide (Human, Rat, Mouse, Porcine, Bovine, Canine, Ovine) Ultra-sensitive (Phoenix Pharmaceuticals, Inc; Karlsruhe, Germany). Fluorescence was determined using an excitation and emission wavelengths of 325 and 420 nm, respectively, in a SpectraMax Gemini EM multiplate fluorescence reader. Results were expressed as pg/mL/mg protein.

Brain cortical insulin levels were measured in 10 μL of each brain cortical homogenate, by using the above mentioned Mercodia Mouse Insulin ELISA Kit. Results were expressed as pg/mL/mg protein.

Brain cortical IGF-1 levels were measured in 5 μL of each brain cortical homogenate (working dilution of 1:20) by the Rat IGF-1 ELISA kit (Biosensis Pty Ltd; Thebarton, South Australia). Absorbance was read at 450 nm, in a SpectraMax Plus 384 microplate reader. Results were expressed as pg/mL/mg protein.

cAMP levels were determined in 7.5 μL of each brain cortical homogenate (working dilution of 1:10) with the cAMP Direct Immunoassay Kit (Colorimetric) (BioVision; Milpitas, CA, USA). Absorbance was read at 450 nm, in a Biochrom Asys Expert 96 UV Microplate Reader (Cambourne, Cambridge, UK). Results were expressed as pmol/mg protein.

Relative PKA activity was determined in 5 μL of each brain cortical homogenate (working dilution of 1:6) by the PKA kinase activity kit (Enzo Life Sciences, Farmingdale, NY, USA). The absorbance was determined at 450 nm, in a SpectraMax Plus 384 multiplate reader. Results were expressed as ng/µL/mg protein.

### Western Blot Analyses

Samples containing denatured brain cortical homogenates (25 μg per lane) were subjected to sodium dodecyl sulphate (SDS)/polyacrylamide gel electrophoresis (SDS/PAGE) (8–15%) and transferred onto polyvinyl difluoride (PVDF) membranes. Then, membranes were blocked for 1 h at room temperature in Tris-buffered saline (TBS, pH 7.4) plus 1 or 5% nonfat dry milk or bovine serum albumine (BSA), plus 0.05% Tween 20. Membranes were then incubated overnight at 4 °C with rabbit IRβ (4B8) (1:1000) or rabbit Phospho-ERK1/2 (Thr202/Tyr204) (1:1000) (Supplementary Fig. 3) primary antibodies (all from Cell Signaling Technology; Leiden, The Netherlands). Membranes were then incubated with the respective anti-rabbit secondary IgG antibodies (1:10,000), for 2 h at room temperature, and developed using ECF. Immunoreactive bands were visualized by the VersaDoc Imaging System (Bio-Rad, Hercules, CA, USA). Fluorescence signal was analysed using the QuantityOne software and the results given as INT/mm^2^. Of note, membranes were then re-probed with the corresponding mouse β-actin (1:5000; from Sigma, St. Louis, MO, USA) or rabbit Total ERK (1:1000; from Cell Signaling Technology; Leiden, The Netherlands) primary antibodies. Results were presented as phosphorylated protein/total protein or protein levels (corresponding to the ratio of each protein vs. β-actin).

### Assessment of Brain Glucose Levels

Brain glucose levels were determined by the above mentioned glucose oxidase method (Infinity Glucose Hexokinase Kit, Thermo Scientific, Middletown, VA, USA), in 3 µL of each brain cortical homogenate. Results were expressed as nmol/L/mg protein.

### Measurement of Brain Glycolysis Markers’ Levels

Pyruvate levels were determined by the Pyruvate Colorimetric/Fluorometric assay kit (BioVision; Milpitas, CA, USA), according to manufacturer’s instructions, in 5–10 µL of each brain cortical homogenate. Fluorescence was determined using an excitation and emission wavelengths of 535 and 590 nm, respectively, in a SpectraMax Gemini EM multiplate fluorescence reader. Results were expressed as pmol/mg protein.

Lactate levels were determined by the Lactate Colorimetric/Fluorometric assay kit (BioVision; Milpitas, CA, USA), according to manufacturer’s instructions, in 5 µL of each brain cortical homogenate. Fluorescence was determined using an excitation and emission wavelengths of 535 and 590 nm, respectively, in a SpectraMax Gemini EM multiplate fluorescence reader. Results were expressed as pmol/mg protein.

### Determination of Brain Adenine Nucleotides’ Levels

Brain cortical homogenates (5 µL/sample, further diluted into 30 µL saline solution) were assayed for adenine nucleotides (ATP, ADP and AMP) by separation in a reverse-phase HPLC, as described by Stocchi *et al*.^60^. The HPLC apparatus was a Beckman-System Gold, consisting of a 126 Binary Pump Model and 166 Variable UV detector controlled by a computer. The detection wavelength was 254 nm, and the column was a Lichrospher 100 RP-18 (5 µm) from Merck (Darmstadt, Germany). An isocratic elution with 100 mM phosphate buffer (KH_2_PO_4_; pH 6.5) and 1.0% methanol was performed with a flow rate of 1 mL/min. The required time for each analysis was 6 min. Adenine nucleotides (ATP, ADP and AMP) were identified by their chromatographic behavior (retention time, absorption spectra, and correlation with standards). Results were presented as µmol/mg protein. Adenylate energy charge was determined according to the following formula: (ATP + 0.5 ADP)/(ATP + ADP + AMP).

### Determination of Brain Triglycerides, Free Fatty Acids (FFA) and Cholesterol Levels

Brain cortical triglycerides levels were determined in 7.5 μL of each brain cortical homogenate with the Triglyceride Quantification Colorimetric/Fluorimetric kit (BioVision; Milpitas, CA, USA).

Brain cortical FFA levels were determined in 7.5 μL of each brain cortical homogenate with the Free Fatty Acid Quantification Colorimetric/Fluorimetric kit (BioVision; Milpitas, CA, USA).

Brain cortical cholesterol levels were determined in 7.5 μL of each brain cortical homogenate with the Total Cholesterol and Cholesteryl Ester Quantification Colorimetric/Fluorimetric kit (BioVision; Milpitas, CA, USA).

Absorbance was read at 570 nm, in a Biochrom Asys Expert 96 UV Microplate Reader (Cambourne, Cambridge, UK). Results of brain cortical triglycerides, FFA and cholesterol levels were expressed as nmol/mg protein.

### Colorimetric/Fluorimetric Evaluation of Initiator and Effector Caspases-Like Activities

Initiator Caspase-1-, Caspase-2-, Caspase-8-, Caspase-9 and Caspase-10-like, and effector Caspase-3- and Caspase-6 like activities were determined colorimetrically using a previously described method, with some modifications^61,62^. Briefly, 5 μL of each brain cortical homogenate (except for Caspase-8-like activity, that required 10 µL of each sample) were incubated at 37 °C, for 2 h, in a reaction medium buffer, containing the following: 25 mM HEPES, 10% (m/v) sucrose and 0.1% (m/v) CHAPS (pH 7.5), supplemented with 10 mM DTT and 100 μM of each specific colorimetric Caspase-like substrate (Ac-YVAD-pNA, Ac-VDAV-pNA, Ac-IETD-pNA, Ac-LEDH-pNA, Ac-AEVD-pNA, Ac-DEVD-pNA and Ac-VEID-pNA). Then, each Caspase-like activity was given by the formation of pNA at 405 nm, in a SpectraMax Plus 384 multiplate reader.

A similar approach was used to determine the activity of Caspase-12-like, by using the Caspase-12 Fluorimetric Substrate, ATAD-AFC (BioVision, Milpitas, CA, USA). Briefly, 5 μL of each brain cortical homogenate were incubated at 37 °C, for 2 h, in the above mentioned reaction medium buffer, supplemented with 10 mM DTT and 50 μM of the caspase-12-like fluorimetric substrate (ATAD-AFC). Then, its Caspase-like activity was given by the cleavage of AFC, using an excitation and emission wavelengths of 400 and 505 nm, respectively, in a SpectraMax Gemini EM multiplate fluorescence reader.

Results were expressed as arbitrary units (a.u.)/mg protein or RFU/mg protein (for Caspase-12-like activity).

### RNA extraction and cDNA synthesis

Total RNA was extracted from brain cortical tissue, using the E.Z.N.A. Total RNA Kit II (Omega bio-tek, Norcross, Georgia, USA) before complementary DNA (cDNA) was synthesized using iScript cDNA Synthesis Kit (Bio-Rad Laboratories, CA, USA), according to manufacturer’s protocol. RNA concentration and purity were measured by a NanoDrop Lite spectrophotometer (Thermo Fisher Scientific, Wilmington, Delaware, USA).

### Real-time quantitative PCR

SsoAdvanced Universal SYBR Green Supermix from Bio-Rad Laboratories was used for RT-qPCR and performed following manufacturer’s protocol. All RT-qPCR plates were run on a CFX96 touch real-time PCR detection system (Bio-Rad, CA, USA). Primers utilized for RT-qPCR validations were designed using either QuantPrime69 or PrimerQuest from Integrated DNA Technologies (http://eu.idtdna.com/PrimerQuest). The efficiency of each primer pair was tested before use by performing a standard curve, and the efficiency criteria for using a primer pair was 90% < E < 110%, with an R^2^ cut-off > 0.990. Housekeeping genes, ATP synthase, H+ transporting, mitochondrial F1 complex, beta polypeptide (*Atp5b*), Calnexin (*Canx*) and Ribosomal Protein L13a (*Rpl13a*) were used for normalization of brain tissue RT-qPCR. Changes in gene expression were calculated using the CFX manager software program (Bio-Rad, CA, USA), using the ΔΔCt method with a fold change cut-off at ≥1.5 and p < 0.05 considered significant. All samples were run in triplicate and relevant positive and negative controls were run on each plate.

### Behaviour Testing

Mice were transported in their home cages to the behavioural testing room and were allowed to acclimate to the room for at least one hour prior to testing.

### Open Field Behaviour Testing

Open field behaviour testing is a way to assess the locomotor and behavioural activity in rodents^63^, which was shown to be affected in the HD mouse model R6/2^64^. Motor activity (distance travelled, mean speed, mean time mobile, number of line crossings and rearing) was evaluated during light cycle in an open field arena with transparent open-topped Plexiglas boxes of 50 × 50 cm, using the Stoelting ANY-MAZE video tracking system (Dublin, Ireland), detecting position of the animal’s head, body and tail. Mice were placed individually in the corner of the open field arena and were recorded for a 30-min period. Data was collected for every 5 minutes.

### Paw Clasping Behaviour Testing

Clasping is a behaviour test that has previously been evaluated in R6/2 mice to study the disease progression, showing a clasping phenotype^32,33^.

Mice were suspended by the tail for 180 s and scored as previously described^65^, from 0 to 2; where 0 represents no clasping behaviour, 1 means that the hind or paws clasp for at least 1 s, and a score of 2 means that the paws clasp for more than 5 s.

### Statistics

GraphPad Prism 6 was used to analyse all data (Graph Pad Software Inc., San Diego, CA, USA). Results are presented as mean ± SEM. After the identification of outliers with the ROUT test and the Kolmogorov-Smirnov normality test, statistical significance was determined using one-way factor analysis of variance (ANOVA), with Bonferroni, Tukey, Sidak or unprotected Fisher’s LSD post-hoc tests (for a Gaussian distribution), or Kruskal-Wallis test, with Dunn post-test (non-Gaussian distribution) for multiple comparisons. Differences with a *P* < 0.05 were considered statistically significant.

### Data availability

All data generated or analysed during this study are included in this published article, and its supplementary dataset.

## Electronic supplementary material

Dataset 1
