# Supplementary material for: Dual Therapy with Liraglutide and Ghrelin Promotes Brain and Peripheral Energy Metabolism in the R6/2 Mouse Model of Huntington’s Disease
Source: Sci Rep. 2018 Jun 12;8:8961. doi: 10.1038/s41598-018-27121-w (PMC5997749; doi:10.1038/s41598-018-27121-w)
Supplement: Supplementary file 1 — Dataset 1 [file 41598_2018_27121_MOESM1_ESM.doc]

**DUAL THERAPY WITH LIRAGLUTIDE AND GHRELIN PROMOTES BRAIN AND PERIPHERAL ENERGY METABOLISM IN THE R6/2 MOUSE MODEL OF HUNTINGTON´S DISEASE**

Ana I Duarte, Marie Sjögren, Maria S Santos, Catarina R Oliveira, Paula I Moreira, Maria Björkqvist

**a.**

**b.**

**c.**

**Supplementary Figure 1.** **Effect of liraglutide plus ghrelin administration on R6/2 mouse body weight and body composition.** Body weight and composition were measured after 2 weeks of liraglutide alone or in combination with ghrelin, or vehicle administration at 12 weeks of age. Significant body weight loss was seen in vehicle treated R6/2 mice compared to WT littermates (a.). Body composition was assessed using DexaScan, in R6/2 mice and WT littermates, and a significant decrease in fat mass was seen in liraglutide treated R6/2 mice compared to WT littermates (b.). There was also a significant decrease in lean mass in vehicle treated R6/2 mice compared to WT littermates (c.). Except for the lower fat mass composition induced by liraglutide alone, the 2-week treatment strategy chosen did not affect R6/2 mouse body weight, or body composition at 12 weeks of age. Data represent mean ± SEM of 10 mice/group, determined by one-way ANOVA with Tukey or Sidak post-hoc test for multiple comparisons. Statistical significance: ^*^*P*<0.05 in Vehicle treated WT mice *vs.* R6/2 liraglutide alone or liraglutide plus ghrelin treated mice.

**a.**

**b.**

**Supplementary Figure 2.** **Effect of liraglutide plus ghrelin administration on R6/2 mouse body weight and food intake along the 2-week treatment.** Effect of co-administration of liraglutide together with ghrelin on R6/2 mouse body weight (a.) and food intake (b.) during treatment was assessed in an additional treatment group. Body weight was monitored twice weekly, starting one week prior to liraglutide and ghrelin administration. Food consumption was monitored daily (from 11 weeks of age) during 2 weeks of liraglutide and ghrelin administration. Data represent mean ± SEM of 8-11 mice/group, determined by two-way ANOVA with Bonferroni post-hoc test for multiple comparisons. Statistical significance: ^*^*P*<0.05, ^**^*P*<0.01 and ^****^*P*<0.0001 in Vehicle-treated WT mice *vs.* R6/2 liraglutide alone or Liraglutide plus ghrelin-treated mice; ^###^*P*<0.001 in Vehicle-treated WT mice *vs* R6/2 liraglutide plus ghrelin-treated mice.


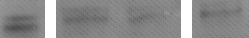


**Anti-P-ERK1,2**

44 kDa

42 kDa


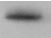

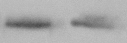

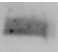


**Anti-total ERK1,2**

44 kDa

42 kDa

**WT**

**NaCl**

**Lira**

**Lira + Ghr**

**R6/2**

**Supplementary Figure 3. Effect of liraglutide plus ghrelin administration on R6/2 mouse brain cortical pivotal downstream signaling pathways to GLP-1, insulin and IGF-1.** Representative Western blot and densitometry of activated ERK1,2 protein levels in cortex of R6/2 mice at 12 weeks and after 2 weeks of treatment with vehicle, liraglutide alone, or co-administration of liraglutide and ghrelin. Data represent mean ± SEM of the indicated number of mice/group.

**Supplementary Table 1. Primer sequences used for gene expression data.**

| **Gene name** | **Forward Sequence (5’ 🡪 3’)** | **Reverse Sequence (5’ 🡪 3’)** |
| --- | --- | --- |
| *Atp5b* | ACCTCGGTGCAGGCTATCTATG | CCAAATGGGCAAAGGTGGTTGC |
| *Canx* | AGCTGTTGAGGCTCATGATGGAC | CTGGAGCTTTGTAGGTGACCTTTG |
| *Caspase 3* | TTGCCAGAAGATACCGGTGGAG | TCCAGGAATAGTAACCAGGTGCTG |
| *Rpl13a* | CCAAAGGTTCCTTAGGCACTGCTC | TGCGCTGTCAGCTCTCTAATGTC |

Table shows primer sequences used for the validation of gene expression data. Efficiency and R^2^ of a standard curve were verified. Efficiency criteria for using a primer pair was 90%<E<110% and the R^2^ cut off was >0.990, with efficiencies below or above being excluded from this study.
